# Letter of John Randolph

**Published:** 1879-03

**Authors:** 


### LETTER OF JOHN RANDOLPH OF ROANOKE.

"My Dear Godson: Although I have so lately written to your better half, and at such tedious length, too, yet I cannot refrain from the attempt to engraft an old head upon young shoulders, notwithstanding my belief that no man was ever the wiser for another's experience. There are too many who are unable to profit by their own; witness the gamester and the spendthrift, not to mention another class of victims of licentious propensities matured into habitual indulgence. Each of these wretched votaries of his darling vice is more sensible of the ruinous effects of his folly than any preacher who exhorts him against the consequences. He that wears the chain best knows how and where the fetter galls. They that declaim against the fixed and rooted ill-habits of their neighbor, under the expectation of reforming him, only show that they themselves are coxcombs. Rather sententious and flat this, you will say, and you will tell the truth.

"Having established yourself in Gloucester, let me remind you 'not to put off until to-morrow what may be done to-day;' and, *a fortiori*, not to leave till next year what could be done this (1830.) Plant all sorts of trees. Man and boy, I knew John Lewis for some forty years or so, although I never was in his house. It is probable he may have saved you the trouble of rearing orchards. But, be that as it may, fail not to have a good apple orchard especially, and banish ardent spirits as a beverage from your table. I have serious fears on this head for a certain young * * *. If, at the beginning, you are obliged to resort to spirits, let your wife make the punch or toddy by measure, of a certain strength never to be increased, according to the good old Virginia fashion.

mistake in pagination

"2. Have no dealings that can possibly be avoided with your neighbors. The disregard of this caution will certainly lead to squabbles and strife.

"3. Take no receipts on loose pieces of paper. Carry a receipt book in your pocket, and take all receipts in it; if you are afraid of losing it, keep it in your desk. Always have the receipts witnessed, when practicable.

"4. Copy, or have copied, all your bills in a book, so that you may at a glance, see the cost of any article or branch of expense.—Without accurate accounts you must fall behind hand. What voyage would a ship make without observation or reckoning? You are now embarked on the voyage of life; without a good look out, you must be cast away.

"5. Form no intimacies with your neighbors under a seven years acquaintance—'*qui vult dicipiatur.*' The rigid observance of my own maxims did not prevent ill blood between some of my neighbors and myself. My maxims preserved me from strife, and from loss by those. With the rest I was on the best of terms.

"6. Economy: the adapting your supplies judiciously to their intended end. This is a gift of God. It cannot be taught; at least, I have tried to learn it all my life, without success. My mother had it in perfection.

"7. Frugality: '*Non intelligunt homines quam magnum vectiga sit parsimonia.*'—It is in the power of every honest man, who means to retain his honesty, to refrain from indulging in expenses which he cannot afford. A disregard of this maxim, the result of their indolent ignorance of their own affairs, has ruined all my name and race; they did not know what they could afford, and some, I fear, did not care.

"I shall send you some acorns of an oak from Turkey, and also a few English. Plant them in beds, keep clean, and transplant at eighteen inches or two feet high. I hope that you will not forget broad-nuts, English, walnuts, (and black walnuts too); filberts, hazlenuts and chestnuts, I will bring or send you. I saw trees of this sort planted about a century ago, at Houghton Hall, by the great Sir R. Walpole, from two to three feet in diameter. This was in August, 1826. They afford a noble shade as well as valuable fruit.

"Get Cobbett's "American Gardener." It can be had at Pishey Thompson's, in Washington. Spare no expense or labor for good enclosures, and tight, warm houses. The parsimony I preach up does not extend to the exclusion of comforts. I hope never to see a fire-place in your house without shovel and tongs and fender; nor with broken windows. When I was on a visit to poor B., he had eight or ten sponging visitors and their horses, and it was with difficulty, that I could get a basin or towel; even the most necessary articles in a bed-chamber were missing. I do not mean the bed; for there was one, although most uncomfortable. No; furnish your rooms well, however plainly. It is a first expense for the whole of your life. Plate and china and glass, you will have no occasion to buy.

"I shall probably never see how you and my darling niece succeeded as house-keepers. Daily and every day, I find that I am sinking. To be laid by the side of my honored parents at Old Matoax, is now the only wish that I have personally to myself. No tomb-stone, no monument for me. Let 'Spring, with dewy fingers cold,' dress the turf that shall cover my no longer feverish head or throbbing heart. If there be any memorial of me, let it be a plain head-stone, with this inscription: 'John Randolph of Roanoke, son of John Randolph of Roanoke, the elder, and Frances Bland, his wife, and stepson of Virginia, born June 2d, 1773, died ——, 1831.' Beyond this last period, I feel that it is impossible, short of a miracle, for my existence to be prolonged. 'Thy will be de done.'

"I am now closely confined to my apartment, with faithful John's aid. I have all the comforts that I am now capable of enjoying; my life hangs upon his.

"I had like to have omitted one special caution against going to the watering places in Autumn in search of health. It is an idle, dissipated, and expensive practice. If you are to live in the lower country, you must accustom yourself to the climate, which I have no hesitation in saying is in every way more healthy than that of the upper country, short of the Alleghany. When I was a boy, agues and fevers were hardly known twenty miles west of the falls of the great rivers.

"The inhabitants of the lower country were always jeered by the trans-mountain people, especially on account of their sickly climate. But now the valley is, perhaps, the sickliest in the State. Who ever heard of the breaking up of old William and Mary by an indigenous plague? In case you should go far enough west (to Montgomery or Wythe) to avoid autumnal disease, you must count upon dying the first time that circumstances oblige you to spend the season at home. There never was complaint of sickness at Warner Hall, until the last Warner introduced the rum fever. The notion of ill health has been a pretext to cover the love of gadding and gossiping; and for a Winter climate, and Spring especially (not to mention roads), there is no comparison.

"Pray mention Mrs. V. Bibber's lowest price for her estate, and John Tabb's also, if practicable.

"Adieu, my children. J. R. of Roanoke.

"To John Randolph Bryan, Esq."

"December 29, 1830.—The weather has been cold (14 deg. of Fahrenheit), but it has moderated. I see that the New York packet of the 1st of this month, has arrived. I received a letter from W. Leigh, of the 21st of November, which must have come by it. I have been more and more unwell the last three days."

"London, Dec. 29, 1830."

